# Unusual Conductance Fluctuations and Quantum Oscillation in Mesoscopic Topological Insulator PbBi_4_Te_7_

**DOI:** 10.1038/s41598-019-43534-7

**Published:** 2019-05-07

**Authors:** Priyanath Mal, Bipul Das, Archana Lakhani, Ganesh Bera, G. R. Turpu, Jong-Ching Wu, C. V. Tomy, Pradip Das

**Affiliations:** 1 Department of Pure and Applied Physics, Guru Ghasidas Vishwavidyalaya, Koni, Bilaspur, C. G. 495009 India; 2 Department of Physics, National Changhua University of Education, Jin-De Road, Changhua, 500 Taiwan; 3 UGC-DAE CSR, University Campus, Khandwa Road, Indore, 452001 India; 4 Department of Physics, Indian Institute of Technology Bombay, Powai, Mumbai, 400076 India

**Keywords:** Electronic properties and materials, Topological insulators

## Abstract

We present a detail study of Shubinikov-de-Haas (SdH) oscillations accompanied by conductance fluctuations in a mesoscopic topological insulator PbBi_4_Te_7_ device. From SdH oscillations, the evidence of Dirac fermions with *π* Berry phase is found and the experimentally determined two main Fermi wave vectors are correlated to two surface Dirac cones (buried one inside the other) of layered topological insulator PbBi_4_Te_7_. We have also found evidence of conductance fluctuations, the root mean square amplitude of which is much higher than the usual universal conductance fluctuations observed in nanometer size sample. Calculated autocorrelation functions indicate periodic unique fluctuations may be associated with the topological surface states in the compound.

## Introduction

The study of the interplay between symmetry and phase transition in condensed matter physics has achieved great importance for several decades. Recently the existence of a new class of material called topological insulators (TIs) has been predicted, where symmetry alone cannot describe its chiral spin polarization at the edge states^1,2^. Angle resolved photoemission spectroscopy (ARPES) measurement in prototype Bi_2_Se_3_^3^ and several other TI single crystal samples^4–6^ demonstrated that an odd number of massless, spin helical Dirac cones (DC) are present at their surface states. Transport^7,8^ and scanning tunneling microscopic^9^ measurements corroborate the spins at the surface states are locked perpendicular to the momenta. In various studies, investigations related to the topological character of these fascinating materials through transport measurements provide some important and novel information e.g., weak antilocalizatoin^10,11^, Shubnikov-de-Hass (SdH) oscillations^12–14^, quantum fluctuations^15,16^, etc. Other than the above phenomena, Checkelsky *et al*.^17^ have observed a unique magneto fingerprint signal in micro-meter size Ca doped Bi_2_Se_3_ single crystal flake at low temperature with angle dependent magnetoresistance (MR) measurement and argued that the novel fluctuations are related to the spin degree of topologically protected surface states. One of the crucial limitation in transport studies to investigate the surface states is the interference from metallic bulk states which hinder the perfect spin polarization at the surface states and the large number of the carrier density that shifts the Fermi level towards the bulk conduction band instead pinning to Dirac point (DP). Moreover, topological insulator materials exposed to the environment also manifest a shift in Fermi level towards the bulk conduction band^18,19^. The surface exposure can be reduced by using inert gas atmosphere, photo resist layer etc., but these involves complications. Therefore, to overcome surface exposure as well as to get maximum surface polarization it is instructive to choose the material very carefully. In search of new TI materials, numerous binary as well as ternary chalcogenides have been investigated along with different doping elements and by applying gate voltages to tune the band gap and the Fermi level^20–26^. Surface and bulk sensitive ARPES studies show that Bi_2_Se_3_ has the largest band gap^3,27^ whereas, PbBi_2_Te_4_ shows the maximum spin polarization (70%)^6^. ARPES studies of PbBi_4_Te_7_ confirm the existence of two Dirac cone, DC1 and DC2, one buried inside the other^28^. The unit cell of PbBi_2_Te_4_ is made by insertion of PbTe layer in Bi_2_Te_3_, consist seven atomic monolayers and similarly, hexagonal unit cell of PbBi_4_Te_7_ single crystal is made by five atomic layers of Bi_2_Te_3_ block and seven-atomic layers of PbBi_2_Te_4_ block, separated by van-der-Waals gap. As crystals used to cleave at the van-der-Waals gap, so, the crystal is terminated either at 5 layers of Bi_2_Te_3_ block or at 7 layers of PbBi_2_Te_4_ block. In the case of 5 layers of Bi_2_Te_3_ terminated surface, the surface sates of PbBi_4_Te_7_ distributed not only at the top 5 layers, but also extended upto next 7 under layers of PbBi_2_Te_4_; unlike 7 layers terminated surface where the surface Dirac cone located solely at the 7 layer^28^. Therefore deep buried topological surface states one inside the other in PbBi_4_Te_7_ receive physical protection without the need of any external efforts. Moreover, due to the existence of large spin polarized surface states in Pb and Bi based alloys we may able to observe clear signature of the magneto finger print as observed by Checkelsky *et al*.^17^, motivated us to study topological insulator PbBi_4_Te_7_. Reduction of the flake thickness of TI single crystals is a powerful tool to elucidate the surface states lead to the fabrication of nano device of PbBi_4_Te_7_ single crystal with thickness 60 nm and length of few micrometer.

Herein, we present for the first time an exploration of the magneto-transport properties of nano device made from mechanically exfoliated flake of PbBi_4_Te_7_ single crystal, synthesized by modified Bridgman method. We present the observations of weak antilocalizatoin, Shubnikov-de-Hass (SdH) oscillations and unique conductance fluctuations. Experimentally determined *π* Berry phase confirmed the Dirac nature of the carriers. The estimated Fermi wave vectors correspond to the two surface Dirac cones (buried one inside other) of PbBi_4_Te_7_. We don’t observe the signature of universal conductance fluctuations (UCF) as expected for the nano device, instead unique conductance fluctuations are observed.

## Result and Discussion

**Figure 1:**
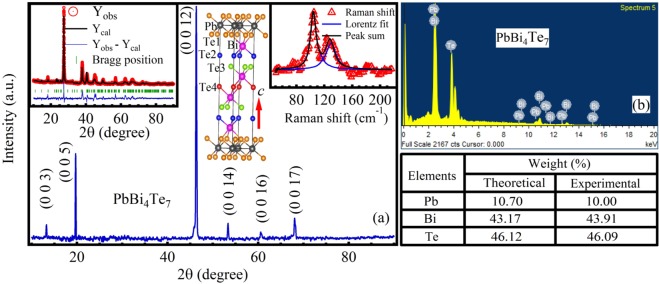
(**a**) X-ray diffraction pattern for the mechanically exfoliated single crystalline plane of PbBi_4_Te_7_, showing (*0 0 l*) growth direction. Inset (Left) illustrates the Rietveld refinement of room temperature XRD data, assures PbBi_4_Te_7_ belongs to P-3m1 space group. Inset (middle) illustrates the three dimensional unit cell of PbBi_4_Te_7_, the frame represents the unit cell. Inset (right) illustrates the room temperature Raman spectra for the mechanically exfoliated single crystal flake for measured in parallel to crystallographic *c* axis. (**b**) EDS spectrum for the single crystal flake, table (bellow) presents the theoretical and experimentally determined weight (%) of the single crystal flake.

Figure 1(a) illustrates the room temperature X-ray diffraction pattern for the mechanically exfoliated flakes of PbBi_4_Te_7_ single crystal. To investigate the growth direction of the crystal, flakes were exfoliated along the lengths of the ingots, for each flake we observed sharp *c*-axis reflections from their Bragg’s planes, inferred the high crystalline quality of the single crystal. To investigate the phase purity of the single crystal, the exfoliated flakes were pulverized in to polycrystalline powder. Rietveld refinement^29^ of the corresponding powder XRD is illustrated in the inset (left panel) of Fig. 1(a) which reveals PbBi_4_Te_7_ belong to P-3m1(164) space group with *a* = *b* = 4.455 Å and *c* = 24.155 Å respectively, good agreement with literature^30–32^. The absence of parasitic peaks confirms the monophasic nature of the single crystal. Refined lattice parameters are modeled^33^ and are illustrated in the inset (middle panel) of Fig. 1(a), reveales the unit cell of PbBi_4_Te_7_ consists of 12 atomic layers of Pb, Bi and Te. In the unit cell, Te-Bi-Te-Bi-Te, five layers atomic block (5 LB) of Bi_2_Te_3_ is separated from seven layer block (7 LB) of Te-Bi-Te-Pb-Te-Bi-Te i.e. PbBi_2_Te_4_ via van-der-Waals gap, the alternate stacking of the 5 LB and 7 LB along *c* direction make the 3D structure of PbBi_4_Te_7_. At room temperature two distinct Raman modes at 104 cm^−1^ and 124 cm^−1^ are identified for the PbBi_4_Te_7_ single crystal for parallel to crystallographic *c* axis measurement. The mode positions are verified by Lorentzian line shape fitting of the modes as illustrated in the inset (right panel) of Fig. 1(a). Figure 1(b) displays the energy dispersive spectrum of X-rays where the experimental atomic weight (%) matches well with the theoretical value.

**Figure 2:**
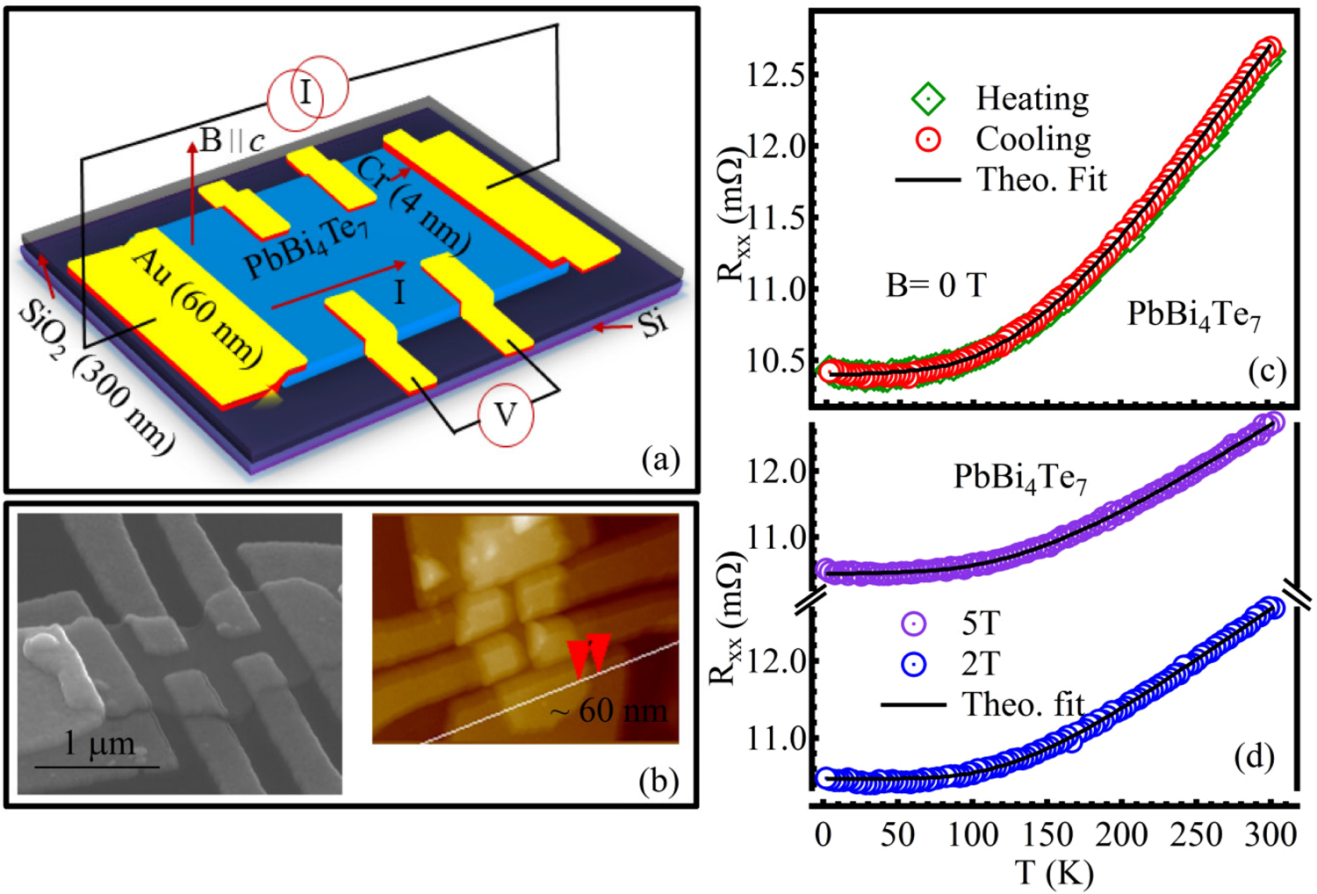
(**a**) Schematic of the PbBi_4_Te_7_ nano device. (**b**) (Right panel) SEM micrograph, (left panel) AFM micrograph of the nano device (flake thickness ~60 nm). Temperature variation of longitudinal resistance of PbBi_4_Te_7_ nano device at (**c**) B = 0 T and (**d**) B = 2, 5 T, respectively; the magnetic field (B) is applied perpendicular to the basal plane of the single crystal and also to the sensing current.

Figure 2(a) represents the schematic of PbBi_4_Te_7_ nano device having flake thickness ~60 nm [see Fig. 2(b)], illustrating the direction of the DC magnetic field applied parallel to the crystallographic *c* axis and perpendicular to the direction of the sensing current. Figure 2(c,d) illustrate the variation of longitudinal resistance (R_*xx*_) as a function of temperature for the nano device at various applied DC magnetic fields (B = 0, 2 and 5 T, respectively) parallel to crystallographic *c* axis. As the temperature decreases, resistance starts to decrease up to ~50 K, with saturating nature bellow it, the behavior is typical for a metallic sample. Temperature dependency of R_*xx*_ can be best described by: ${R}_{xx}={R}_{0}+\beta {e}^{-\frac{\theta }{T}}+\gamma {T}^{2}$ where, R_0_ is the residual resistance of the crystal. The exponential and quadratic terms represent electron-phonon and electron-electron interactions, respectively. R_0_ = 0.01042 Ω, *β* = 0.00916 Ω, *θ* = 527.56 K and *γ* = 7.779 × 10^−9^ Ω K^−2^ corresponded to the best fit for B = 0 T curve. The fitted parameters indicate the conduction at higher temperatures is assisted by electron-phonon scattering mainly whereas, the electron-electron interaction can be neglected. The determined phonon frequency is $(\omega =\frac{{K}_{B}\theta }{\hslash }) \sim 6.90\times {10}^{13}$ rad s^−1^. In the presence of magnetic field (B = 2 T and 5 T), the R_0_ value does not change considerably; R_0_ = 0.0104(7) Ω for B = 2 T and R_0_ = 0.0104(4) Ω for B = 5 T, respectively as illustrated in Fig. 2(d), suggests absence of magnetic or spin dependent impurities in the crystal. The residual resistivity ratio (RRR) for B = 0 T, $\frac{\rho (300\,K)}{\rho (2\,K)}$ = 1.21, is of the order of TI single crystals reported previously^34,35^.

**Figure 3:**
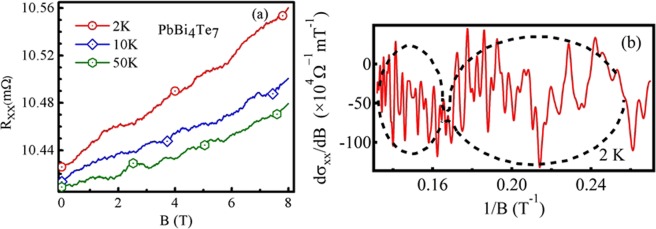
(**a**) Field (B) variation of R_*xx*_ at 2, 10 and 50 K, respectively. (**b**) Oscillatory components of $\frac{d{\sigma }_{xx}}{dB}$ at 2 K, showing beating nature; the dotted black lines illustrates the beat envelopes.

**Figure 4:**
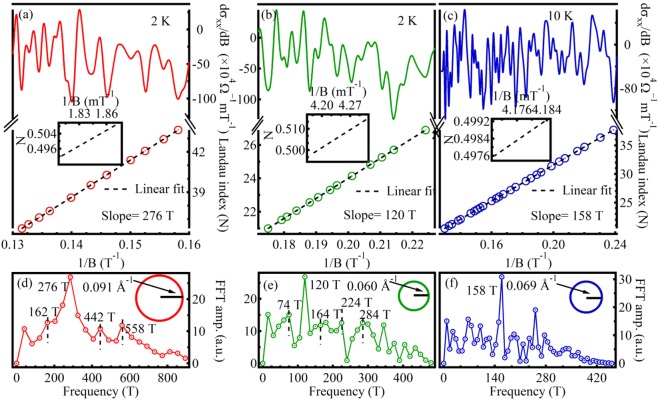
SdH oscillations for different ranges of applied magnetic field at (**a,b**) 2 K and (**c**) 10 K, respectively. Lower panels illustrate Landau level fan diagrams, ascertain ~*π* Berry phase of the carriers. Insets illustrate extrapolations to Y-axis of the linear fits having slopes equal to the frequencies of the highest intense FFT peaks of different field ranges. (**d**–**f**) Fast Fourier transform (FFT) spectra of the oscillations in the respective upper panel field ranges. The schematic Fermi circles are illustrated in the insets without considering any warping effect.

**Figure 5:**
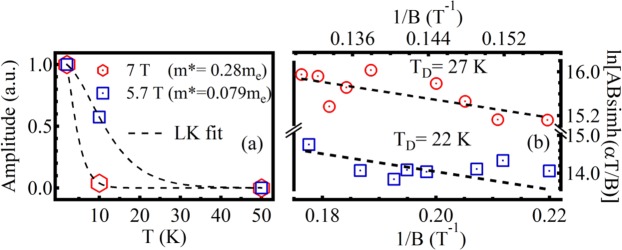
(**a**) Lifshitz-Kosevich fit of the temperature dependent normalized amplitudes of oscillation. (**b**) Dingle plots for (lower st. line) DC1 and (upper st. line) DC2, respectively at 2 K.

Figure 3(a) illustrates field variation of magnetoresistance (R_*xx*_(B)) at different temperatures with a magnetic field applied perpendicular to the basal plane of the crystal. The common features of the curves are fluctuations along with oscillations in the entire range of magnetic field and non-saturating enhancement of magnetoresistance upto highest applied field (8 T). The reproducibility of the oscillatory features of the curves at different temperatures make it distinct from the random noise. To investigate the effect of magnetic field on the resistance of PbBi_4_Te_7_ nano device, magnetoresistance curves are smoothed using first order polynomial^36^. The subtraction of the smoothed background of the actual signal gives the fluctuations, the nature of which discussed in detail later. The smoothed curves as illustrated in Fig. 3(a), reveals an oscillatory nature of R_*xx*_. For better contrast, derivative of the longitudinal conductivity $({\sigma }_{xx}=\frac{1}{{\rho }_{xx}})$ derived from the smoothed signal (resistance) is shown in Fig. 3(b) $(\frac{d{\sigma }_{xx}}{dB}\,{\rm{v}}{\rm{s}}\,\frac{1}{B}\,{\rm{for}}\,2\,{\rm{K}})$. We see here increasing amplitude of oscillation with decreasing $\frac{1}{B}$ value, which is a clear signature of the SdH effect. A careful observation reveals that the oscillations show a beating nature corresponding to the presence of multiple frequencies related to different Fermi surfaces. To understand the contribution of each Fermi surface towards transport properties of the crystal, we have divided oscillatory pattern into different regions depending on the node to node distance of the beats. At higher $\frac{1}{B}$ values and outside the marked beating envelopes do not show considerable amplitudes. SdH oscillations are the manifestation of oscillatory nature of density of states when Landau levels pass through Fermi energy and become depopulated upon varying the magnetic field B. When Fermi energy is in between two consecutive Landau levels, the Landau levels below the Fermi surface are completely filled, leaving zero density of states at the Fermi energy. As a consequence, we observe minima of conductivity. The SdH oscillations can be expressed as:

$$\cos [2\pi (\frac{{f}_{SdH}}{B}-\frac{1}{2}+\gamma )]$$

where, *f*_*SdH*_ be the frequency of oscillations, B be the applied magnetic field and *γ* be the phase factor. The fast Fourier transform (FFT) of the oscillations at 2 K reveles most prominent frequencies at 276 and 120 T possessing highest amplitudes (Fig. 4(d,e)) and at 158 T for 10 K ambient temperature (Fig. 4(f)). Dividing oscillations for a particular temperature in different regions, FFT spectrum reveals several other frequencies along with the most prominent frequencies. An insight towards the FFT reveals, peaks at higher fields show smaller amplitudes with frequencies that are near integer multiple of the peak frequencies observed at low field ranges. For instance, the observed hump like 162 T and most prominent 276 T peaks as marked in Fig. 4(d) are also present in FFT signal corresponds to higher field range [in Fig. 4(e)] with modulated frequencies centered at 164 and 284 T, respectively. The observed other frequencies [in Fig. 4(d)] at 442 (74 × 5.97, 224 × 1.97) and 558 T (284 × 1.96) are near integer multiple of frequencies 74, 224 and 284 T frequencies, respectively. Thus the Fermi surface of lower K_*F*_ value also contribute towards the conductivity at high field but suppressed by the higher order Fermi surface which contains comparatively large number of carriers. In order to investigate topological phase by means of the observed FFT frequencies i.e., whether they are trivial or non-trivial topological states, Landau levels are indexed at the amplitude minima of $\frac{d{\sigma }_{xx}}{dB}$ vs $\frac{1}{B}$ plots and the corresponding fan diagrams for all the field ranges are plotted [see Fig. 4(a,b) for 2 K and Fig. 4(c) for 10 K oscillations]. The fan diagrams are linearly fitted well with the slope of respective frequencies obtained from the FFT (Fig. 4(d–f)), for each of the corresponding field ranges. The extrapolations of the fitted lines intercept the Y-axes [landau index (N)] closely at ${\rm{Y}}=\frac{1}{2}$ [inset of Fig. 4(a–c)], gives a measure of Berry phase. The intercept *γ* related to Berry phase as^14^: $\gamma =\frac{{\varphi }_{B}}{2\pi }$ where, *ϕ*_*B*_ is the Berry phase acquired by the carriers upon moving around the Dirac point. The determined Berry phase is ~*π*, confirming the non-trivial nature of Dirac fermions of the topological surface states. The Fermi wave vectors (for 2 K) correspond to the observed frequencies at 276 T and 120 T (the two most prominent frequencies) are determined from Onsagar’s relation: ${f}_{SdH}=(\frac{\hslash }{2\pi e})\pi {{K}_{F}}^{2}$ and are found to be of ~0.091 Å^−1^ (say, K_*F*2_) and 0.060 Å^−1^ (say, K_*F*1_), respectively. The estimated K_*F*_ value for 158 T (at 10 K), ~0.069 Å^−1^, is nearly equal to the K_*F*2_ observed at 2 K. In order to determine the effective mass of electrons, amplitude variation of oscillations with temperature is fitted with Lifshitz-kosevich (LK) factor^37^: ${F}_{LK}=\frac{{\rm{\Gamma }}T}{sinh({\rm{\Gamma }}T)}$, ${\rm{\Gamma }}=\frac{2{\pi }^{2}{K}_{B}}{\hslash {\omega }_{c}}$ and *ω*_*c*_ is the cyclotron frequency and related to the effective mass (m*) as: ${\omega }_{c}=\frac{eB}{{m}^{\ast }}$. The calculated effective masses from ourdata in Fig. 5(a) are 0.28 m_*e*_ (K_*F*2_) and 0.079 m_*e*_ (K_*F*1_) at field values ~7 T and ~5.7 T, respectively, m_*e*_ is the free electron mass. The corresponding Fermi velocity, *v*_*F*_ $(=\frac{\hslash {K}_{F}}{{m}^{\ast }})$ are 3.76 × 10^5^ ms^−1^ (K_*F*2_) and 8.80 ×10^5^ ms^−1^ (K_*F*1_), respectively. The estimated position of Fermi energy level from Dirac point of K_*F*2_ is $(\,=\,{m}^{\ast }{{v}_{F}}^{2})$ ~185 meV, which corresponds to the binding energy ~300 meV of the reported ARPES for PbBi_4_Te_7_ ^28^. The ratio of the wave vectors (and respective absolute values) corresponding to the observed frequencies at 276 T and 120 T is close to the reported ratio (and close to the respective absolute values) of DC2 and DC1 at binding energy 300 meV^28^, indicating K_*F*1_ and K_*F*2_ correspond to topological surface states of DC1 and DC2, respectively. ARPES reveled shape of the constant energy contours for DC1 shows more energy dependency than DC2 i.e. DC1 shows more warping effect of Fermi surface over DC2^28^. The warping of Fermi surface leads to peak splitting as illustrated in Fig. 4(a–c). Carrier lifetime (*τ*) is determined from the field dependence of SdH oscillations for a fixed temperature i.e. from Dingle analysis of two different regions (corresponds to each beat) of 2 K data. Figure 5(b) illustrates the semilog plot of ^34^ D = ABsinh$(\frac{\alpha T}{B})$ vs $\frac{1}{B}$, the slope of which gives Dingle temperature (T_*D*_); A is the amplitude of oscillation determined by averaging the vertical distance of crests from the adjacent valleys and *α* = $\frac{14.7{m}^{\ast }}{{m}_{e}}$ TK^−1^. The Dingle temperatures (T_*D*_) correspond to DC1, DC2 are 22 and 27 K, respectively, obtained from the slopes of the ln(D) vs $\frac{1}{B}$, plots give best fit. The estimated carrier life time (*τ*) from T_*D*_ values $({T}_{D}=\frac{\hslash }{2\pi {K}_{B}\tau })$ are 5.5 × 10^−14^ s (DC1) and 5.98 × 10^−14^ s (DC2), respectively. The obtained mean free paths *l*_*SdH*_(=*v*_*F*_*τ*) of the carriers are 48.4 nm (DC1) and 22 nm (DC2), respectively. The mobility *μ*_*SdH*_ $(=\frac{e\tau }{m\ast })$ of the carriers are 1229 (DC1) and 375 (DC2) cm^2^ V^−1^ s^−1^, respectively. The high value of *μ*_*SdH*_ and low effective masses of DC1, DC2 confirms their surface origin. The estimated *μ*_*SdH*_ value for DC2 is small compared to DC1 and quite above the bulk mobility of prototypes TIs^38^, reflects the DC2 is deep buried inside the DC1. At 2 K the estimated surface carrier densities n_*s*_ $(=\frac{\pi {K}_{F}^{2}}{{(2\pi )}^{2}})$ are 2.86 × 10^12^ cm^−2^ for DC1 and 6.58 × 10^12^ cm^−2^ for DC2.

**Figure 6:**
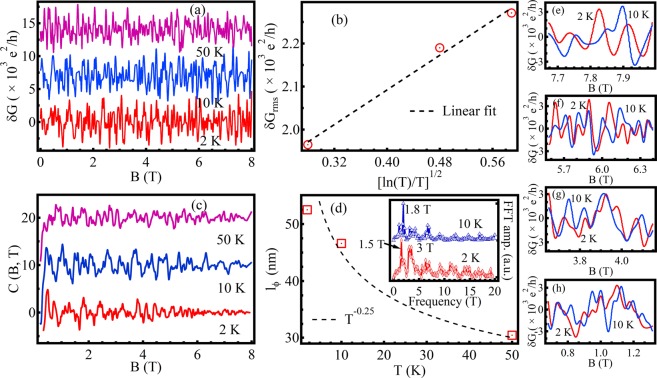
(**a**) Magneto conductance at T = 2, 5 and 10 K, respectively. The curves are shifted vertically for clarity. (**b**) *δ*G_*rms*_ as a function of temperature illustrate their linear dependency. *δ*G_*rms*_ shows comparatively large values over $\frac{{e}^{2}}{h}$ confirms the nature of the oscillations not to be of universal conductance fluctuations type. (**c**) Field variation of correlation function C(B, T) at different temperatures, showing oscillatory nature. (**d**) Power law decay of l_*ϕ*_ with temperature is obtained from periods of oscillations of C(B, T). Inset in (**d**) illustrates the FFT spectrum of correlation function. (**e–h**) Zoomed in regions of (**a**).

We now focus our attention on the conductance fluctuations obtained by subtraction of the smoothed background of the actual magnetoresistance signal. At low temperatures, universal conductance fluctuations (UCF) are particularly significant in single crystalline nanoflakes when the thickness of the flake is comparable to the phase coherence length of the charge carriers. The UCF arises from interference of electrons paths around the defect centers upon sweeping the magnetic field^39^. To explore the possibility whether the fluctuations observed are UCF, we plot absolute *δ*G $(G=\frac{1}{{R}_{xx}})$ as a function of magnetic field in Fig. 6(a) for three representative temperatures, where *δ*G is background subtracted conductance. Zoomed in regions of Fig. 6(a) are illustrated in Fig. 6(e–h), showing repeatability of *δ*G at different temperatures. It can be seen that the absolute conductance fluctuations are nominally depends on magnetic field in the entire range of magnetic field at different temperatures. To quantify the observed fluctuations, *δ*G_*rms*_ [see Fig. 6(b)] is determined as^40^ ${[\langle {(\delta G-\langle \delta G\rangle )}^{2}\rangle ]}^{\frac{1}{2}}$, where 〈…〉 be the ensemble averaging. A decaying fluctuations with increasing temperature is evident from Fig. 6(b) where a ${(\frac{ln(T)}{T})}^{\frac{1}{2}}$ dependence of *δ*G_*rms*_ is observed, indicating the surface origin of conductance fluctuations^41^ The decreasing fluctuations amplitude upon increasing the temperature contemplates us to consider the transport model of UCF proposed by Lee *et al*.^42^. But, the very high value of *δ*G_*rms*_ compared to the universal value $\sim \frac{{e}^{2}}{h}$ discard the presence of UCF in our nano device. To investigate the origin of fluctuations, we have plotted field variation of the autocorrelation function^17^ C(B, T) = $\frac{\langle \delta G(B,T)\delta G(B+{\rm{\Delta }}B,T)\rangle }{\langle \delta {G}^{2}\rangle }$, to give a measure how does a pair of peaks at two different field values are correlated. Figure 6(c) shows the variation of correlation function at 2 K with the applied field sweep. There is no evidence of power law decay, moreover some periodic patterns are observed. The highest peaks (B_*s*_) in FFT of the correlation function obtained are 1.5 T and 3 T (2^*nd*^ harmonic of 1.5 T), respectively at 2 K. For 10 K it, is at 1.8 T [see inset of Fig. 6(d)]. The l_*ϕ*_ value at each temperature are smaller than the flake thickness (60 nm) is another evidence of the surface origin of conductance fluctuations. The oscillations decay with increasing field, which is same as observed by Checkelsky *et al*.^17^, and exactly opposite to the observation we have for the SdH oscillations. The characteristics length, l_*ϕ*_ $(=\sqrt{\frac{{\varphi }_{0}}{{B}_{s}}})$ corresponds to the observed frequencies decreases exponentially according to *l*_*ϕ*_ ~ *T*^−0.25^ [see Fig. 6(d)]. In the light of above discussion, we reckon that the fluctuations observed in the present study may be analogous to the observed fluctuations in Ca doped Bi_2_Se_3_ by Checkelsky *et al*.^17^, arising due to spin degree of topological surface states, though the angle-dependent conductivity measurement in future is necessary to relate the observed fluctuations with spin degree of surface states. Here it is noteworthy that the large fluctuations superimposed on the background of magnetoresitivity data are not affecting the topological surface states with non-trivial *π* Berry phase, attributed the robustness of its topological phase.

In conclusion, we report the Shubinikov-de-Haas oscillations and conductance fluctuations in a mesoscopic topological insulator PbBi_4_Te_7_ device. The Shubinikov-de-Haas oscillations corroborate two unambiguous transport signature, first, it provides evidence of surface Dirac fermions with *π* Berry phase and secondly, the determined two wave vectors by FFT analysis are closely related to the two surface Dirac cones (buried one inside the other) of the layered topological insulator PbBi_4_Te_7_. Though, it is natural to observe Universal conductance fluctuations in nanometer size sample yet, the root mean square amplitude is inconsistent with UCF. The field variation of autocorrelation function of the fluctuations indicate the observed fluctuations may like the magnetofingerprint observed in Ca doped Bi_2_Se_3_ crystal^17^.

## Methods

Single crystal of layered chalcogenide PbBi_4_Te_7_ was grown by melting the stoichiometric homogeneous mixture of Bi, Pb and Te of analytical grade with purity >99.99% in an evacuated quartz tube at 1223 K, the ampule was then cooled down to 893 K at a rate of 2 Khr^−1^. followed by furnace cooling to room temperature. The crystal quality and the phase identification were carried out through room temperature x-ray diffraction (XRD) data collected by a 9 kW Rigaku X-ray diffractometer, operated at 3 kW, equipped with Cu-K_*α*_ radiation in the 2*θ* range of 10° to 90°. Chemical homogeneity was confirmed by the elemental analysis of the energy dispersive spectrum (EDS) of X-rays recorded with Carl Zeiss EVO10 SEM system with an Oxford X-ray detector. Room temperature Raman spectra were collected with Technos STR-500 micro-Raman spectrometer equipped with 532 nm diode laser source having spectral resolution of 1 cm^−1^. To avoid surface oxidation, the laser power was kept at <2 mW. Nano device was made using an exfoliated flake of uniform thickness ~60 nm (confirmed by atomic force microscopy) on clean Si/SiO_2_ wafers (with SiO_2_ layer thickness ~300 nm) by e-beam lithography. Electrical contact pads were made by thermal deposition of Cr (4 nm)/Au (60 nm) bi-layers followed by lift-off where exposures of the flakes to air were minimized by using inert gas atmosphere. In plane electronic transport measurements were carried out with AC transport option of Physical Property Measurement System (PPMS) (Quantum design Inc., USA) using a dc magnetic field applied perpendicular to the sensing current and along the crystallographic *c* (00*l*) direction.
